# Long-Chain Fatty Acids Degradation by *Desulfomonile* Species and Proposal of “*Candidatus* Desulfomonile Palmitatoxidans”

**DOI:** 10.3389/fmicb.2020.539604

**Published:** 2020-12-17

**Authors:** Joana I. Alves, Andreia F. Salvador, A. Rita Castro, Ying Zheng, Bart Nijsse, Siavash Atashgahi, Diana Z. Sousa, Alfons J. M. Stams, M. Madalena Alves, Ana J. Cavaleiro

**Affiliations:** ^1^Centre of Biological Engineering, University of Minho, Braga, Portugal; ^2^Laboratory of Microbiology, Wageningen University & Research, Wageningen, Netherlands; ^3^Laboratory of Systems and Synthetic Biology, Wageningen University & Research, Wageningen, Netherlands

**Keywords:** *Desulfomonile*, palmitate, sulfate reduction, long-chain fatty acids, organohalide respiration, metagenome

## Abstract

Microbial communities with the ability to convert long-chain fatty acids (LCFA) coupled to sulfate reduction can be important in the removal of these compounds from wastewater. In this work, an enrichment culture, able to oxidize the long-chain fatty acid palmitate (C_16__:__0_) coupled to sulfate reduction, was obtained from anaerobic granular sludge. Microscopic analysis of this culture, designated HP culture, revealed that it was mainly composed of one morphotype with a typical collar-like cell wall invagination, a distinct morphological feature of the *Desulfomonile* genus. 16S rRNA gene amplicon and metagenome-assembled genome (MAG) indeed confirmed that the abundant phylotype in HP culture belong to *Desulfomonile* genus [*ca.* 92% 16S rRNA gene sequences closely related to *Desulfomonile* spp.; and *ca*. 82% whole genome shotgun (WGS)]. Based on similar cell morphology and average nucleotide identity (ANI) (77%) between the *Desulfomonile* sp. in HP culture and the type strain *Desulfomonile tiedjei* strain DCB-1^T^, we propose a novel species designated as “*Candidatus* Desulfomonile palmitatoxidans.” This bacterium shares 94.3 and 93.6% 16S rRNA gene identity with *Desulfomonile limimaris* strain DCB-M^T^ and *D. tiedjei* strain DCB-1^T^, respectively. Based on sequence abundance of *Desulfomonile*-morphotype in HP culture, its predominance in the microscopic observations, and presence of several genes coding for enzymes involved in LCFA degradation, the proposed species “*Ca.* Desulfomonile palmitatoxidans” most probably plays an important role in palmitate degradation in HP culture. Analysis of the growth of HP culture and *D. tiedjei* strain DCB-1^T^ with short- (butyrate), medium- (caprylate) and long-chain fatty acids (palmitate, stearate, and oleate) showed that both cultures degraded all fatty acids coupled to sulfate reduction, except oleate that was only utilized by HP culture. In the absence of sulfate, neither HP culture, nor *D. tiedjei* strain DCB-1^T^ degraded palmitate when incubated with *Methanobacterium formicicum* as a possible methanogenic syntrophic partner. Unlike *D. tiedjei* strain DCB-1^T^, “*Ca.* Desulfomonile palmitatoxidans” lacks reductive dehalogenase genes in its genome, and HP culture was not able to grow by organohalide respiration. An emended description of the genus *Desulfomonile* is proposed. Our study reveals an unrecognized LCFA degradation feature of the *Desulfomonile* genus.

## Introduction

Long-chain fatty acids (LCFA) are carboxylic acids containing more than 12 carbon atoms. LCFA are the main products of lipids hydrolysis, and are important contaminants in diverse industrial wastewater (Alves et al., 2009). These hydrophobic compounds can inhibit microbial growth, and are generally associated with sludge flotation and foaming in conventional aerobic and anaerobic wastewater treatment systems (Chipasa and Mędrzycka, 2006; Alves et al., 2009). Sulfate is also commonly found in some lipid/fatty acid-containing wastewater, such as food-processing, slaughterhouses and edible oil refineries (Sousa et al., 2009a; Hao et al., 2014). Therefore, exploring the activity of sulfate-reducing microorganisms, that use sulfate as terminal electron acceptor in the degradation of organic compounds, represent an interesting alternative for LCFA removal.

In the domain Archaea, only one sulfate-reducing archaeon (*Archaeoglobus fulgidus*) was reported to oxidize LCFA (Khelifi et al., 2010). In the Bacteria domain, 17 sulfate-reducing species are described as LCFA-degraders. These are distributed by 16 genera and 5 different families, namely *Desulfobacteraceae*, *Desulfarculaceae*, *Desulfohalobiaceae*, *Syntrophobacteraceae*, and *Peptococcaceae*, within the phyla *Proteobacteria* and *Firmicutes* (Rabus et al., 2006; Sousa et al., 2009a, 2010). From those, 15 species can oxidize saturated LCFA (without double bonds in the aliphatic chain) up to 16 carbon atoms (C_16__:__0_, palmitate), and 9 of these 15 species also grow with stearate (C_18__:__0_). The ability to use unsaturated LCFA coupled to sulfate reduction was only confirmed for *Desulfatibacillum aliphaticivorans* (Cravo-Laureau et al., 2004) and *Desulfatiferula oleofinivorans* (Cravo-Laureau et al., 2007), both belonging to *Desulfobacteraceae* family. These two species are thus the only sulfate-reducing bacteria (SRB) described as capable of growing with oleate (C_18__:__1_) or linoleate (C_18__:__2_). Some SRB oxidize LCFA completely to CO_2_, while other convert LCFA to acetate, in both cases coupled to sulfate reduction to sulfide (Sousa et al., 2010). LCFA-degrading SRB are also known to grow on short- and medium-chain fatty acids (SCFA and MCFA), which contain less than 6 and 6–12 carbon atoms, respectively (Muyzer and Stams, 2008; Sousa et al., 2009a; Hao et al., 2014). The microbiology of SRB involved in fatty acids (SCFA, MCFA and LCFA) degradation was reviewed by Sousa et al. (2010).

Under sulfate-reducing conditions, SRB may also utilize hydrogen or acetate (which are intermediaries of LCFA degradation), working as syntrophic partners of LCFA-oxidizing proton-reducing acetogenic bacteria (Sousa et al., 2009b, 2010). For example, *Desulfovibrio* sp. G11 is a SRB that works as hydrogen-scavenger during butyrate oxidation by *Syntrophomonas saponavida* (Wu et al., 2007) or during butyrate, caproate and caprylate degradation by *Syntrophus aciditrophicus* (Jackson et al., 1999). Acetate is used as substrate by several SRB, e.g., species from *Desulfobacter*, *Desulfobacterium*, *Desulforhabdus*, and *Desulfobacca* genera (Sousa et al., 2010). In the absence of sulfate, some SRB are capable of oxidizing organic compounds in syntrophy with a hydrogenotrophic methanogenic partner [e.g., glycerol degradation by *Desulfovibrio alcoholovorans* in co-culture with *Methanospirillum hungatei* (Qatibi et al., 1991)], but for LCFA this has never been reported.

Long-chain fatty acids-oxidizing SRB have been retrieved from oil field waters and from marine and freshwater sediments, but not from anaerobic sludge or anaerobic bioreactors treating LCFA (Sousa et al., 2009a). Considering that the majority of the microorganisms in anaerobic sludge are unknown or are poorly characterize with regard to LCFA degradation with sulfate, this largely unexplored diversity of anaerobic sludge encloses a high biotechnological potential. Therefore, expanding current knowledge on the microorganisms involved in LCFA degradation with sulfate is important.

The aim of this work was to study microbial communities with the ability to degrade LCFA coupled to sulfate reduction, as well as to identify microbial key players involved in these processes and isolate novel microorganisms. We enriched a sulfate-reducing culture from anaerobic sludge, with the ability to degrade palmitate, and identified a novel species. This species and its closest relatives were further tested for its ability to degrade SCFA, MCFA, and LCFA coupled to sulfate reduction, or under methanogenic conditions in syntrophy with a hydrogenotrophic methanogen.

## Materials and Methods

### Enrichment of a Palmitate-Degrading Sulfate-Reducing Culture (HP Culture)

A highly enriched culture degrading 1 mM sodium palmitate (≥98.5%, Sigma-Aldrich) with 20 mM sodium sulfate, was obtained after more than ten successive transfers (10% v/v inoculum). This enriched culture will be further designated as HP culture. The inoculum was anaerobic granular sludge from a brewery wastewater treatment plant (Porto, Portugal) [5 g wet weight (w.w.), with 0.08 g of volatile solids per g w.w.] that was incubated with 1-hexadecene (0.6 mM, ≥99%, Sigma-Aldrich) and sulfate (20 mM) for three successive transfers. A bicarbonate-buffered mineral salt medium was used (Stams et al., 1993), containing (per liter): KH_2_PO_4_, 0.41 g; Na_2_HPO_4_⋅2H_2_O, 0.53 g; NH_4_Cl, 0.3 g; CaCl_2_⋅2H_2_O, 0.11 g; MgCl_2_⋅6H_2_O, 0.10 g; NaCl, 0.3 g; NaHCO_3_, 4.0 g; resazurin, 0.05 g; 1 mL of acid and alkaline trace element stock solutions each, and 0.2 mL of vitamin stock solution. Trace elements and vitamins were prepared as described previously (Stams et al., 1993). The medium (50 mL medium) was dispensed into 120 mL serum bottles, and the bottles’ headspace was exchanged and pressurized with N_2_/CO_2_ (80:20% v/v, 170 kPa). Before inoculation, medium was reduced with sodium sulfide (Na_2_S⋅9H_2_O, 0.8 mM final concentration). Substrates and electron acceptors were added to the medium from sterile stock solutions before inoculation. All cultures were prepared in duplicate and incubated statically at 37°C in the dark. Palmitate consumption, sulfide formation and microbial community composition were monitored. Using the same experimental procedure, additional culturing strategies, such as serial dilutions and soft agar cultures, different electron donors (palmitate, 1 mM; H_2_/CO_2_, 80:20% v/v, 170 kPa; acetate, 20 mM; pyruvate, 20 mM; butyrate, 10 mM; hexadecane, 1 mM; 1-hexadecene, 1 mM) and acceptors (sulfate, 20 mM; fumarate, 20 mM; nitrate, 10 mM; 3-chlorobenzoate, 1 mM; 2-chlorobenzoate, 1 mM; 3-bromobenzoate, 1 mM) were used to characterize the HP culture and to attempt the isolation of dominant microorganisms within the microbial community (Supplementary Figure 1).

### Fatty Acids Degradation by Members of the *Desulfomonile* Genus

The HP culture and *Desulfomonile tiedjei* strain DCB-1^T^ (DSM 6799, obtained from The German Collection of Microorganism and Cell Cultures DSMZ, Braunschweig, Germany) were tested for their ability to degrade the following fatty acids: butyrate (C_4__:__0_, 10 mM), caprylate (C_8__:__0_, 5 mM), palmitate (C_16__:__0_, 1 mM), stearate (C_18__:__0_, 1 mM) or oleate (C_18__:__1_, 1 mM) [all used as sodium salts (purity ≥ 98.5%)]. All the substrates were dissolved in water, but for palmitate, stearate and oleate, the stock solution was heated in a water batch at 60–80°C. With this procedure we were able to completely dissolve the LCFA, and thus transfer the correct amount of substrate from the sterile stock solution to each serum bottle of the assay. The substrates were tested separately, and were added to the serum bottles before inoculation, using sterile syringes and needles. Culture medium was prepared as described in section “Enrichment of a Palmitate-Degrading Sulfate-Reducing Culture (HP Culture).” Sodium sulfate (20 mM) was added as the electron acceptor. To provide optimal growth conditions, based on the work of DeWeerd et al. (1990), both cultures were supplemented with hemin (50 μg L^–1^) and 1,4-naphthoquinone (200 μg L^–1^). Yeast extract (1 g L^–1^) was also added to the *D. tiedjei* strain DCB-1^T^ cultures according to the DSMZ *Desulfomonile* medium (medium 521; DSMZ, Braunschweig, Germany). A transfer of 10% (v/v) of each pre-grown culture was done to initiate the incubations. HP culture was pre-grown with palmitate, 1 mM, and *D. tiedjei* strain DCB-1^T^ was pre-grown with pyruvate, 20 mM, both with sulfate, 20 mM. Those transfers (as well as all the others in this work) were made based on the following criteria: more than 90% of the substrate was consumed (assessed indirectly by the amount of sulfide produced, considering the stoichiometry of the reactions, Table 4); at least 15 to 20 days of incubation were passed, ensuring the inoculation at an active growth stage of the culture; and an optical density (OD at 600 nm) of 0.3–0.4 for both cultures. The assays were prepared in triplicate and incubations were conducted statically, at 37°C and in the dark. Consumption of fatty acids and formation of sulfide, acetate and other SCFA were monitored during the experiments. The cultures were visually checked for turbidity (growth) and periodically examined by phase contrast microscopy. To test organohalide respiration with LCFA, HP culture and *D. tiedjei* strain DCB-1^T^ were incubated with palmitate (1 mM) and 3-chlorobenzoate (3-CB) as the electron acceptor (1 and 2 mM), in the absence of sulfate. Incubations of HP culture with 3-CB and H_2_/CO_2_ (80:20% v/v, 170 kPa) were also performed. Reductive dehalogenation of 2-chlorobenzoate (2-CB) and 3-bromobenzoate (3-BB) (1 mM) by HP culture was further tested with either palmitate (1 mM) or H_2_/CO_2_ (80:20% v/v, 170 kPa).

### Assessment of Potential Syntrophic Relationships Between *Desulfomonile* Species and a Hydrogen Consuming Methanogen

*Methanobacterium formicicum* (DSM 1535^T^) was obtained from DSMZ (Braunschweig, Germany) and pre-grown in a mineral media prepared as described by Sousa et al. (2013) with H_2_/CO_2_ (80:20% v/v, 170 kPa). Before inoculation of *D. tiedjei* strain DCB-1^T^ or HP culture (10% v/v inoculum), bottles’ headspace was exchanged and pressurized with N_2_/CO_2_ (80:20% v/v, 170 kPa), using a portable Bunsen burner and sterile needles and filters (0.2 μm), to remove traces of CH_4_ and H_2_. The bottles were subsequently amended with 1 mM of palmitate. The assays were done in triplicate and were incubated statically, at 37°C and in the dark. Methane production was measured during the incubations.

### Microscopy

Phase contrast micrographs were obtained with an Olympus CX41 RF microscope (Tokyo, Japan) and an Olympus Altra 20 image acquisition system. Scanning electron microscopy (SEM) was performed using a SEM FEI Nova 200 (FEG/SEM) equipment (FEI, Hillsboro, OR, United States). Samples for SEM observations were prepared as described elsewhere (Salvador et al., 2017).

### Analytical Methods

Short-chain fatty acids (C2 to C6) were analyzed by liquid chromatography (HPLC) as described previously (Paulo et al., 2018). MCFA and LCFA (C8 up to C18) were measured by gas chromatography (GC Varian 3800, Agilent, Santa Clara, CA, United States) after esterification with propanol and extraction with dichloromethane, as described by Neves et al. (2009). Total dissolved sulfide was measured using LCK653 cuvette tests (Hach-Lange GmbH, Düsseldorf, Germany) and a DR 2800 spectrophotometer (Hach-Lange GmbH, Düsseldorf, Germany). Samples were collected with minimum contact with air, transferred to a zinc acetate solution (2% w/v) prepared with 0.2 ml/L of concentrated acetic acid, and immediately analyzed. Methane formation was measured by gas chromatography (Chrompack 9000) equipped with a FID detector, as described before (Paulo et al., 2018). Halogenated benzoates and benzoate were analyzed by HPLC, with a Poroshell 120 EC-C18 column (Agilent, Santa Clara, CA, United States), using a three-step gradient profile consisting of: (i) 90% eluent A (0.1% formic acid in water) and 10% eluent B (0.1% formic acid in acetonitrile) for 2 min, (ii) 90–20% eluent A and 10–80% eluent B for 14 min and hold at 20% eluent A and 80% eluent B for 3 min, (iii) followed by 20–90% eluent A and 80–10% eluent B for 1 min. Components were detected with a UV detector at 210 nm.

### 16S rRNA Gene Sequencing and Phylogenetic Analysis

20 mL of HP culture in a highly enriched phase (after more than 20 successive transfers growing on palmitate), were used for microbial community analysis. DNA extraction was performed using the FastDNA SPIN kit for soil (MP Biomedicals, Solon, OH, United States) according to the manufacturer’s protocol. 16S rRNA gene amplification were done by using two universal primer’s set: Bact27-f/Uni1492-r for Bacteria and Arch109-f/Uni1492r for Archaea (Lane, 1991; Grosskopf et al., 1998). Sequencing analysis by Illumina MiSeq platform, as well as taxonomic bioinformatics data analysis, were performed as described previously (Salvador et al., 2019). The nucleotide sequences were deposited in the European Nucleotide Archive (ENA) under accession number PRJEB26656. Cloning and sequencing of the nearly complete sequence of the 16S rRNA gene were performed as previously described (Alves et al., 2013). Sanger sequencing was done at Macrogen (Amsterdam, Netherlands), and the obtained 16S rRNA gene sequence (1373 bp) was deposited at ENA under the accession number LS453291. This sequence was compared to 16S rRNA gene sequences from other sulfate-reducing fatty acid degrading bacteria, and represented in a phylogenetic tree. The tree was calculated with the MEGA X software package (Kumar et al., 2018), using the Neighbor-Joining method (Saitou and Nei, 1987) and the p-distance method (Nei and Kumar, 2000).

### Metagenome Sequencing, Binning and Annotation

Genomic DNA from HP culture (30 mL) growing on palmitate and sulfate was isolated using a Gram-positive genomic DNA isolation kit (MasterPure Gram positive DNA purification Kit, Epicenter, Madison, WI, United States). DNA quality was checked by electrophoresis in a 0.8% (w/v) agarose gel. The metagenome of HP culture was sequenced using Illumina HiSeq X Ten platform (Illumina Inc., San Diego, CA, United States) by Novogene (Beijing, China). Assembly was performed with SPAdes (v3.13.0) using default parameters except for -k 91 and–only assembler (Bankevich et al., 2012). The metagenomic binning Metawrap v1.2 (docker version) was used with the binning tools MaxBin2, MetaBat2, and Concoct (Alneberg et al., 2014; Wu et al., 2016; Uritskiy et al., 2018; Kang et al., 2019). Binning results from the three binners where used as an input for the bin refinement module in Metawrap which resulted in 4 bins. These 4 bins where used as an input for the reassembled bins module of Metawrap. The final reassembled bins where used to calculate the relative abundances of the bins in the data. Bins were checked for completion and contamination according to the methodology described by Bowers et al. (2017), and further taxonomic affiliation was done based on Bowers et al. (2017). Functional annotation of the bins was performed using the Rapid Annotation Subsystem Technology (RAST) (Aziz et al., 2008) and Prokka (Seemann, 2014). Additional functional annotation was performed to obtain further information on the proteins involved in the fatty acid degradation pathway in HP culture (4 different bins) and in *D. tiedjei* strain DCB-1^T^. Protein sequences were annotated with reference to the Clusters of Orthologous Groups (COG) database (with reCOGnizer, version 1.2.3, available at http://www.github.com/iquasere/reCOGnizergithub.com/iquasere/reCOGnizer and also at http://www.anaconda.org/bioconda/recognizeranaconda.org/bioconda/recognizer, which also assigns proteins to EC numbers), and to the Conserved Domain Database by using the NCBI CDD web search tool (Marchler-Bauer and Bryant, 2004). Average nucleotide identity (ANI) of genome bins obtained from HP culture and *D. tiedjei* strain DCB-1^T^ was calculated using JSpecies Web Server^1^ (Richter et al., 2016). Raw sequencing data, primary assembly and assembled bins were deposited in the ENA at EMBL-EBI under accession number PRJEB35900.

## Results and Discussion

**FIGURE 1:**
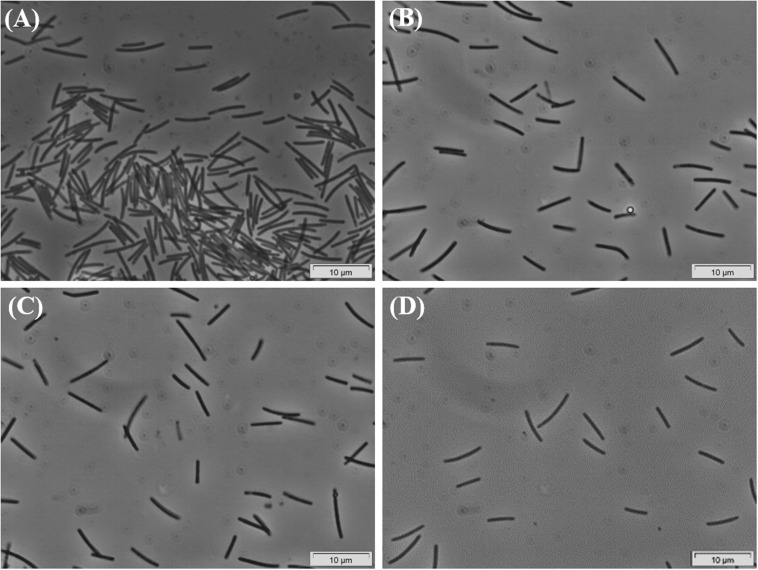
Phase-contrast micrographs of HP culture growing in palmitate, showing the predominance of the *Desulfomonile*-morphotype. Images **(A–D)** were taken during the enrichment process, over 5 years. Bar, 10 μm. Due to overexposure by the camera, all images had to be manipulated by changing color saturation to 0, decreasing the light (−20%) and increasing the contrast (+20%).

**FIGURE 2:**
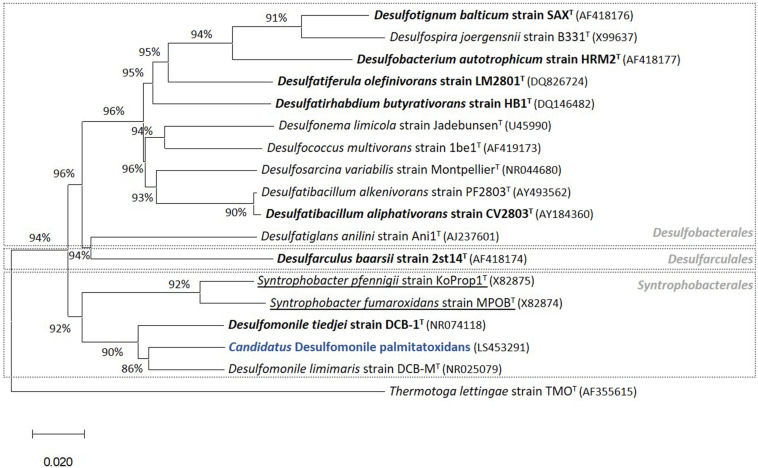
Phylogenetic tree of 16S rRNA gene sequences highlighting (in blue) the position of “*Candidatus* Desulfomonile palmitatoxidans” from HP culture relative to other species of the same genus, as well as selected reference sequences of SRB within the class *Deltaproteobacteria*. These SRB, belonging to the *Desulfobacterales*, *Desulfarculales*, and *Syntrophobacterales* orders, were chosen based on their capability to degrade SCFA, MCFA, and/or LCFA (Sousa et al., 2007b, 2009b, 2010). LCFA degraders are shown in bold and syntrophs are underlined. The phylogenetic tree was calculated using the MEGA X software package (Kumar et al., 2018). The evolutionary history was inferred using the Neighbor-Joining method (Saitou and Nei, 1987). The optimal tree with the sum of branch length = 0.97544146 is shown. The evolutionary distances were computed using the p-distance method (Nei and Kumar, 2000). GenBank accession numbers of 16S rRNA gene sequences are indicated in parentheses. *Thermotoga lettingae* strain TMO^T^ (AF355615) was used as an outgroup. Bar, 2 % estimated difference in nucleotide sequence position.

Successive transfers of an anaerobic culture on palmitate and sulfate resulted in a highly enriched culture (designated by HP culture), predominantly composed of a bacterium assigned to *Desulfomonile* genus. The dominance of the *Desulfomonile*-morphotype within the palmitate-enriched culture was always clearly observed through microscope observations performed over almost 5 years (Figure 1). Microbial community analysis using partial 16S rRNA amplicon sequencing showed that *Desulfomonile* sp. represented 91.5 ± 0.5% of the total bacterial community (Table 1). This bacterium is closely related to *Desulfomonile limimaris* strain DCB-M^T^ and to *D. tiedjei* strain DCB-1^T^, sharing 94.3 and 93.6% 16S rRNA gene identity, respectively (Table 1 and Figure 2). Archaeal community was not analyzed because methane was not produced during the incubations and because we failed to amplify 16S rRNA genes using archaeal universal primers. Four metagenome-assembled genomes (MAG) were obtained from HP culture among which the MAG related to *Desulfomonile* showed the highest relative abundance (*ca*. 82%) (Table 2), which is in line with the 16S rRNA gene sequencing analysis and microscopic observations. These line of evidence support the conclusion that the HP enrichment culture is dominated by *Desulfomonile* sp.

**TABLE 1:** Microbial community composition of HP culture.

Taxonomic identification at family^(a)^/genus^(a)^ level	Relative abundance (%)^(b)^	Closest cultured relatives based on 16S rRNA gene identity	16S rRNA gene identity (%)	Accession number
*Syntrophaceae/Desulfomonile*	91.5 ± 0.5	*Desulfomonile limimaris* strain DCB-M^(c)^	94^(c)^	NR025079
*Synergistaceae/Aminivibrio*	7.5 ± 0.5	*Aminivibrio pyruvatiphilus* strain 4F6E^(d)^	100^(d)^	NR113331
*Syntrophobacteraceae/Desulforhabdus*	0.9 ± 0.2	*Desulforhabdus amnigena* strain ASRB1^(d)^	100^(d)^	NR029289
*Desulfovibrionaceae/Desulfovibrio*	0.2 ± 0.1	*Desulfovibrio* sp. SRL8083^(d)^	98^(d)^	FJ841984

*(a) Classified using the RDP Naïve Bayesian Classifier. (b) Percentage calculated based on the total number of sequence counts obtained by Illumina sequencing, which was 17629 and 15664 (duplicate sequencing reactions). (c) Results of sequence alignment by using BLAST against the NCBI nucleotide database of nearly complete 16S rRNA gene sequence (1373 bp; results from cloning and sequencing). (d) Results of sequence alignment by using BLAST against the NCBI nucleotide database of partial 16S rRNA gene sequences (approximately 291 bp obtained from Illumina amplicon sequencing).*

**TABLE 2:** Metagenome-assembled genomes (MAG) from the HP culture.

Genomic Bins	Relative abundance (%)	Taxonomy (at family level)	Genome Size (bp)	Completeness (%)	Contamination (%)
Bin 1	5.3	*Syntrophobacteraceae*	4114016	93.2	2.4
Bin 2	81.7	*Syntrophaceae*	5958014	96.3	1.3
Bin 3	7.7	*Desulfovibrionaceae*	3269345	91.4	0.3
Bin 4	5.3	*Synergistaceae*	3091291	100	0.0

Both 16S rRNA amplicon sequencing and whole genome shotgun (WGS) showed the presence of other microorganisms assigned to *Synergistaceae*, *Desulfovibrionaceae*, and *Syntrophobacteraceae* families (Tables 1, 2). These microorganisms were less abundant in HP culture, when compared with the relative abundance of the organisms assigned to *Desulfomonile* sp. Based on 16S rRNA gene sequence analysis, they were affiliated to *Aminivibrio*, *Desulforhabdus*, and *Desulfovibrio* genera, representing approximately 8.5% of the total community, based on 16S rRNA amplicon sequencing, and 18% based on WGS (Tables 1, 2). None of the genera present in HP culture was previously associated to LCFA degradation. *Desulforhabdus amnigena* strain ASRB1^T^ (showing 100% 16S rRNA gene identity to the *Desulforhabdus* identified in HP culture, Table 1) is known to use hydrogen and acetate simultaneously, formate, propionate and butyrate (Oude Elferink et al., 1995), but is unable to grow with palmitate or oleate (Sousa et al., 2009a). The presence of SRB affiliated to *Desulfovibrio* genus in enrichment cultures grown on oleate or palmitate and sulfate was previously reported and linked to hydrogen consumption (Sousa et al., 2009a). Members of the *Desulfovibrio* genus have also been detected in oleate-degrading cultures without sulfate, although their role in these enrichments was not clear (Sousa et al., 2010; Salvador et al., 2019). Furthermore, a *Desulfovibrio* sp. isolated from an oleate-degrading enrichment culture failed to degrade oleate coupled to sulfate reduction (Salvador et al., 2019). Microorganisms assigned to *Aminivibrio*, and particularly *Aminivibrio pyruvatiphilus*, typically grow on amino acids and were never reported as fatty acid degraders (Honda et al., 2013).

Members of the *Desulfomonile* genus occur naturally in different sulfidogenic environments, such as petroleum reservoirs (Gao et al., 2015), oily sludge (Ma et al., 2017), acidic environments (Koschorreck et al., 2010; Sanchez-Andrea et al., 2012; Hausmann et al., 2016) and municipal wastewater (Biswas et al., 2014). Thus far, only three *Desulfomonile* species have been described, namely *D. tiedjei* strain DCB-1^T^, *D. limimaris* strain DCB-M^T^ and *Desulfomonile* sp. strain IA6 (DeWeerd and Suflita, 1990; DeWeerd et al., 1990; Sun et al., 2001; Rios-Hemandez, 2003). *D. tiedjei* strain DCB-1^T^ and *D. limimaris* strain DCB-M^T^ can grow on butyrate (SCFA) with thiosulfate or 3-chlorobenzoate as electron acceptor, respectively (DeWeerd et al., 1990; Sun et al., 2001). Although the most abundant microorganism in HP culture is affiliated to the *Desulfomonile* genus, the ability of these microorganisms to utilize MCFA and LCFA has not been reported so far. However, other bacteria, relatively closed related to the *Desulfomonile* sp. in HP culture, namely *Desulfatibacillum alkenivorans* and *D. aliphaticivorans* (showing about 88% 16S rRNA gene identity, Figure 2), are described as fatty acids-degrading SRB. *D. aliphaticivorans* is even described as a SRB with the ability to degrade SCFA, MCFA and LCFA (both saturated and unsaturated) (Cravo-Laureau et al., 2004).

Several attempts were made to isolate the *Desulfomonile* species from the HP culture, to test its ability to degrade palmitate in pure culture. Serial dilutions and successive transfers combined with different electron donors and acceptors, in liquid and solid medium are among the strategies applied (Supplementary Figure 1), but the three other morphotypes, although in minor abundance, could not be eliminated from the culture.

**FIGURE 3:**
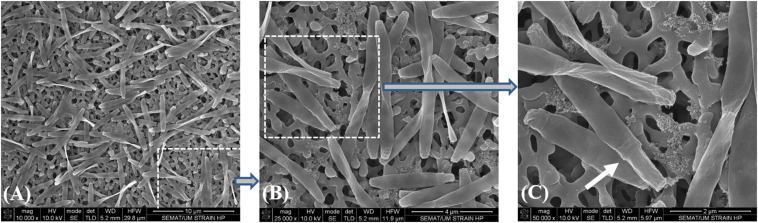
Sequential microscope observations of HP culture, dominated by *Desulfomonile*-morphotype: **(A)** SEM micrographs–Bar, 10 μm; **(B)** SEM micrograph–Bar, 4 μm; and **(C)** SEM micrograph–Bar, 2 μm. The white arrow in panel **(C)** indicates the collar structure of the cell.

Due to the impossibility to isolate this species, all the incubations with SCFA, MCFA and LCFA were performed with HP culture. In order to investigate if the LCFA-degrading ability is a common feature of the *Desulfomonile* genus, *D. tiedjei* strain DCB-1^T^ was tested as well. *D. limimaris* strain DCB-M^T^ is no longer available in culture collections and *Desulfomonile* sp. strain IA6 was never deposited. These bacteria are also not available at the laboratories where they were isolated (James Tiedje and Joseph Suflita, personal communication) and hence could not be included in this study. The type strain *D. tiedjei* strain DCB-1^T^ showed 77% ANI to the MAG of *Desulfomonile* species in HP culture. Based on the ANI cut-off value of 94% for the definition of a new species (Richter and Rossello-Mora, 2009; Richter et al., 2016), we propose a novel species designated “*Candidatus* Desulfomonile palmitatoxidans.” “*Ca.* Desulfomonile palmitatoxidans” showed morphological characteristics typical from *Desulfomonile* species, namely rod shaped cells (Figure 3) and an invagination of the cell wall known as collar structure (DeWeerd et al., 1990; Sun et al., 2001) that is highlighted in Figure 3C. The cell size (0.6 μm by 5–6 μm), inability to form spores and lack of motility are additional characteristics similar to the other known *Desulfomonile* strains.

**FIGURE 4:**
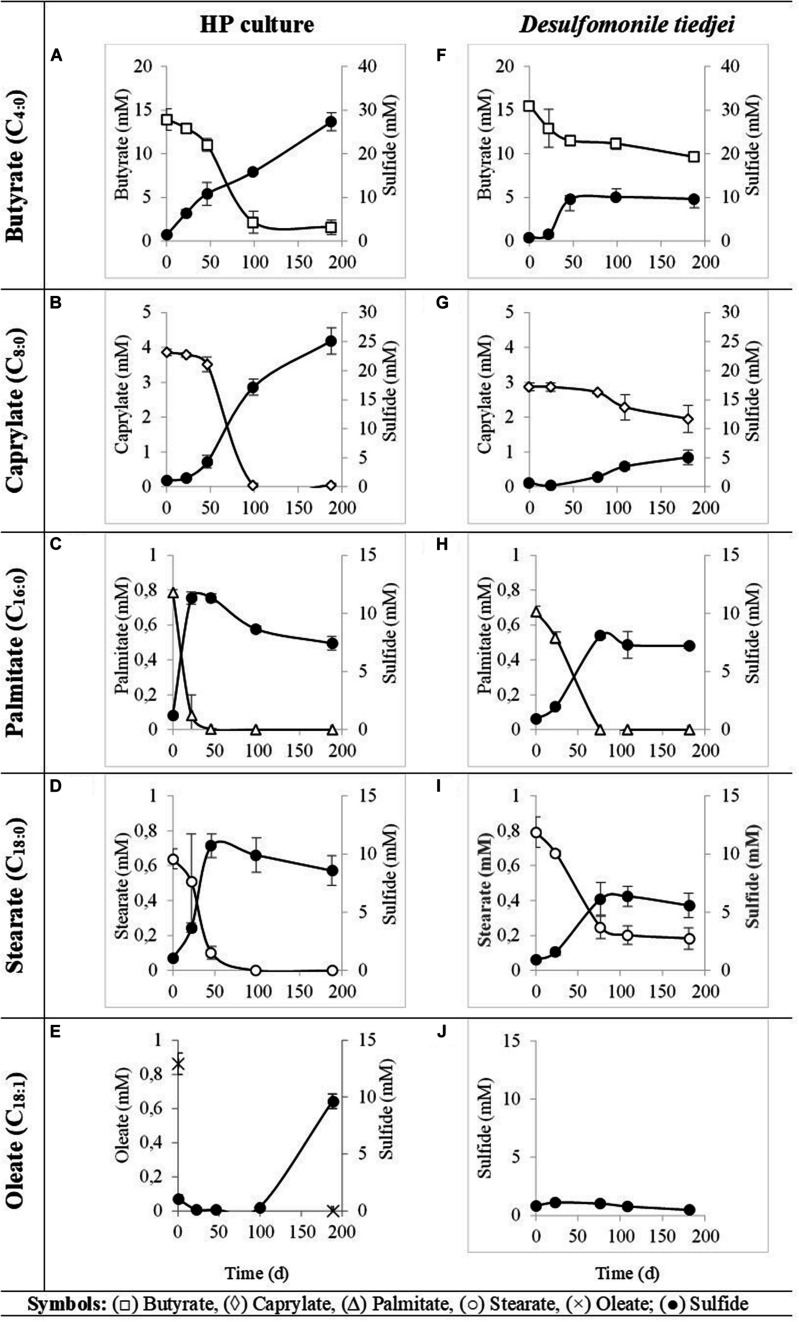
Fatty acids degradation (open symbols) coupled to sulfate reduction (measured as sulfide production, closed symbols) by HP culture **(A–E)** and *D. tiedjei* strain DCB-1^T^ **(F–I)**. Oleate was not measured in panel **(J)**. The bars indicate standard deviations at each time point (*n* = 3).

HP culture degraded all fatty acids tested (Figures 4A–E and Table 3) with correspondent sulfate reduction (Table 4), assessed indirectly by the amount of sulfide produced (Figures 4A–E and Table 5). Palmitate and stearate (LCFA, 1 mM each) were completely degraded within 22 and 46 days of incubation, respectively (Figures 4C,D). In contrast, longer incubations, of around 99 days, were necessary for complete caprylate (MCFA, 5 mM) degradation, and for the conversion of 89 ± 5% of the added butyrate (SCFA, 10 mM) (Figures 4A,B and Table 5). Oleate (unsaturated LCFA, 1 mM) was only degraded after a lag phase of more than 100 days (Figure 4E). From the amount of substrate degraded, the total sulfide formed and the theoretical sulfide value that could be expected from the substrate degraded (considering the stoichiometry of the reactions, Table 4), electron recovery was calculated and varied between 84 and 112% in these assays (Table 5). Similar to the incubations with palmitate, microscopic observations showed predominant growth of *Desulfomonile*-morphotype within HP culture when growing with butyrate, caprylate, stearate and oleate, suggesting an involvement of “*Ca.* Desulfomonile palmitatoxidans” in fatty acids degradation.

**TABLE 3:** Comparison of physiological and morphological characteristics between (a) HP culture, (b) *D. limimaris* strain DCB-M^T^, and (c) *D. tiedjei* strain DCB-1^T^.

Characteristics	(a) HP culture*	(b) *D. limimaris*	(c) *D. tiedjei*
Origin	Anaerobic granular sludge	Marine sediment	Sewage sludge
DNA G + C content	55% (for “*Ca.* Desulfomonile palmitatoxidans”)	nt	49%
Fermentative growth (with pyruvate)	−	−	+
**Electron acceptors:**			
	(with palmitate)	(with lactate)	(with pyruvate)
Sulfate	+	+	+
Thiosulfate	nt	+	+
Fumarate	−	+	nt
Nitrate	−	+	nt
**Electron donors:**			
H_2_/CO_2_	+ (with sulfate)**	+ (with 3-CB)	+ (with sulfate)
Formate	nt	+ (with 3-CB)	+ (with sulfate)
Pyruvate	+ (with sulfate)**	+ (with 3-CB)	+ (with sulfate)
Acetate	+ (with sulfate)**	− (with 3-CB)	− (with sulfate) ± (with thiosulfate)
Butyrate	+ (with sulfate)***	+ (with 3-CB)	± (with sulfate)* ± (with thiosulfate)
Caprylate	+ (with sulfate)***	nt	+ (with sulfate)*
Palmitate	+ (with sulfate)***	nt	+ (with sulfate)*
Stearate	+ (with sulfate)***	nt	+ (with sulfate)*
Oleate	+ (with sulfate)***	nt	− (with sulfate)*
Hexadecane	− (with sulfate)	nt	− (with sulfate)*
Hexadecene	− (with sulfate)	nt	− (with sulfate)*
**Halobenzoates as electron acceptors:**			
3-CB	− (with palmitate) − (with H_2_/CO_2_) − (with pyruvate) nt (with formate) nt (with lactate) nt (with butyrate) nt (with propionate)	nt (with palmitate) + (with H_2_/CO_2_) + (with pyruvate) + (with formate) + (with lactate) + (with butyrate) + (with propionate)	− (with palmitate)* − (with H_2_/CO_2_) + (with pyruvate) − (with formate) nt (with lactate) nt (with butyrate) nt (with propionate)
2-CB	− (with palmitate) nt (with lactate)	nt (with palmitate) − (with lactate)	nt (with palmitate) nt (with lactate)
3-BB	− (with palmitate) nt (with lactate)	nt (with palmitate) + (with lactate)	nt (with palmitate) nt (with lactate)

Data are from DeWeerd et al. (1990); Sun et al. (2001) and this study. (*) Data from this study. (**) It is unclear whether “*Ca.* Desulfomonile palmitatoxidans” or the partner bacteria oxidize the substrate. (***) Microscopic observations give evidence of predominant growth of *Desulfomonile*-morphotype within HP culture when growing with these substrates. 3-CB, 3-chlorobenzoate; 2-CB, 2-chlorobenzoate; 3-BB, 3-bromobenzoate. Symbols: +, utilized; −, not utilized; ±, poorly utilized; nt, not tested.

**TABLE 4:** Sulfate reduction reactions and Δ*G*^0^′ for butyrate (C_4__:__0_), caprylate (C_8__:__0_), palmitate (C_16__:__0_), stearate (C_18__:__0_), and oleate (C_18__:__1_) oxidation.

FATTY ACID	REACTION	Δ*G*^0^′ (kJ/reaction)^(a)^
Butyrate	C_4_H_7_O_2_^–^ + 2.5 SO_4_^2–^ → 4 HCO_3_^–^ + 2.5 HS^–^ + 0.5 H^+^	−123.1
Caprylate	C_8_H_15_O_2_^–^ + 5.5 SO_4_^2–^ → 8 HCO_3_^–^ + 5.5 HS^–^ + 1.5 H^+^	−268.7
Palmitate	C_16_H_31_O_2_^–^ + 11.5 SO_4_^2–^ → 16 HCO_3_^–^ + 11.5 HS^–^ + 3.5 H^+^	− 560.2
Stearate	C_18_H_35_O_2_^–^ + 13 SO_4_^2–^ → 18 HCO_3_^–^ + 13 HS^–^ + 4 H^+^	−633.0
Oleate	C_18_H_33_O_2_^–^ + 12.75 SO_4_^2–^ + H_2_O → 18 HCO_3_^–^ + 12.75 HS^–^ + 4.25 H^+^	−675.7

*(a) Gibbs free energy changes at standard conditions (solute concentrations of 1 mol L^–1^, gas partial pressure of 10^5^ Pa, *T* = 25°C, pH 7). Calculations were done based on the values presented by Thauer et al. (1977).*

**TABLE 5:** Fatty acids degradation and electron recovery in the assays with HP culture or *D. tiedjei* strain DCB-1^T^.

FATTY ACID	HP Culture		*D. tiedjei*	
	Degradation (%)	Electron recovery (%)^(a)^	Degradation (%)	Electron recovery (%)^(a)^
Butyrate	89 ± 5	84 ± 9	38 ± 2	78 ± 11
Caprylate	99 ± 2	87 ± 8	32 ± 8	116 ± 8
Palmitate	100 ± 0	112 ± 2	100 ± 0	80 ± 3
Stearate	100 ± 0	93 ± 10	76 ± 11	90 ± 10
Oleate	100 ± 0	94 ± 4	–	–

*(a) Electron recovery = total sulfide formed/theoretical sulfide value. The theoretical sulfide value was calculated considering the amount of substrate degraded and the stoichiometry of the reactions (Table 4).*

*Desulfomonile tiedjei* strain DCB-1^T^ degraded almost all fatty acids tested (Figures 4F–I), although always slower than HP culture (Figure 4 and Table 5) and it was unable to degrade oleate, even in long term incubations (more than 150 days). Whereas faster LCFA degradation could be a result of the acclimation of HP culture during long-term enrichments using palmitate, a similar pattern was also observed with SCFA and MCFA. For example, HP culture and *D. tiedjei* strain DCB-1^T^ produced the same amount of sulfide from butyrate (around 35% of the theoretical value expected from complete conversion to CO_2_, Table 4) after 46 and 77 days of incubation, respectively (Figures 4A,F), showing that butyrate degradation by HP culture was significantly faster. Furthermore, *D. tiedjei* strain DCB-1^T^ degraded only 38 ± 2% and 32 ± 8% of the added butyrate and caprylate, respectively, while for HP culture, degradation of these two substrates was almost complete (Table 5). *D. tiedjei* strain DCB-1^T^ was previously reported to grow poorly with butyrate coupled with thiosulfate reduction (DeWeerd et al., 1990; Table 3). Considering the amount of substrate degraded by *D. tiedjei* strain DCB-1^T^, 78–116% of the electrons formed were recovered as sulfide from the substrates tested (Table 5).

The newly described ability of *Desulfomonile* species to degrade LCFA is supported by the high number of gene copies coding for enzymes involved in LCFA degradation that could be found both in *D. tiedjei* strain DCB-1^T^ and in “*Ca.* Desulfomonile palmitatoxidans” genomes. Both bacteria have several gene copies coding for key enzymes necessary for complete fatty acid degradation, i.e., a total of 85 and 96 in *D. tiedjei* strain DCB-1^T^ and in “*Ca.* Desulfomonile palmitatoxidans” genomes, respectively. Detailed functional analysis can be found in Supplementary Tables 1, 2.

In particular, in “*Ca.* Desulfomonile palmitatoxidans” genome, we found 32 gene copies of long-chain-fatty-acid-CoA ligase (FadD), 25 copies of acyl-CoA dehydrogenases (FadE), 11 copies of enoyl-CoA hydratase, 6 copies of 3-hydroxyacyl-CoA dehydrogenase (FadB), and 17 copies for acetyl-CoA acetyltransferases (FadA) (Supplementary Table 1).

Genes coding for beta-oxidation enzymes (the LCFA biodegradation pathway) were also found in other MAG obtained from HP culture (Supplementary Table 3). However, genes for complete fatty acid beta-oxidation were not found in the MAG assigned to *Synergistaceae* and *Desulfovibrionaceae*. On the contrary, the *Syntrophobacteraceae*-MAG contained the genes for the complete beta-oxidation pathway (which is in line with the fact that this species degrades SCFA, namely propionate and butyrate), including two genes coding for fatty acid CoA ligases that were long-chain specific. This shows the genomic potential of *Desulforhabdus* sp. to perform LCFA degradation. However, as mentioned before, *D. amnigena* strain ASRB1^T^ could not degrade neither palmitate, nor oleate under sulfate reducing conditions (Sousa et al., 2009a). Given the high taxonomic proximity of the microorganism assigned to *Syntrophobacteraceae* in HP culture and *D. amnigena* strain ASRB1^T^ (100% 16S rRNA gene identity, Table 1), these results suggest that this bacterium was not the main palmitate degrader in HP culture. Although the ability of this microorganism to degrade LCFA cannot be discarded, because of its genomic potential, the much higher abundance of *Desulfomonile*-like cells comparatively to the other morphotypes in HP culture, during incubation with LCFA, point that *Desulfomonile* sp. was the key player in this process. The fact that another *Desulfomonile* species, *D. tiedjei* strain DCB-1^T^, is able to degrade LCFA, whereas *Desulforhabdus* species were never reported as LCFA degraders, also corroborate this hypothesis.

For both *Desulfomonile* cultures, in all the conditions tested, acetate or other possible intermediary fatty acids were not detected in the liquid medium, which suggests that LCFA are completely degraded to CO_2_. However, because “*Ca.* Desulfomonile palmitatoxidans” is in HP culture together with other microorganisms, one cannot discard that in this culture LCFA can undergo partial oxidation, forming H_2_ and acetate that can then be utilized by other partner bacteria. Nevertheless, this is not the case for *D. tiedjei* strain DCB-1^T^.

Organohalide respiration was previously reported as a metabolic characteristic of *Desulfomonile* genus, although apparently strain IA6 does not show this ability (Rios-Hemandez, 2003). The two described *Desulfomonile* species were reported to conserve energy for growth from organohalide respiration using pyruvate as an electron donor and halobenzoates as the electron acceptors (DeWeerd and Suflita, 1990; DeWeerd et al., 1990; Sun et al., 2001). In addition to pyruvate, *D. limimaris* strain DCB-M^T^ also oxidizes lactate, formate, H_2_, butyrate and propionate coupled to reductive dehalogenation (Sun et al., 2001). Nevertheless, the ability to perform organohalide respiration using LCFA as electron donors was never reported. In this study, experiments with palmitate were done shifting the final electron acceptor from sulfate to 3-CB, but neither HP culture nor *D. tiedjei* strain DCB-1^T^ showed dehalogenation activity (Table 3). Control experiments with *D. tiedjei* strain DCB-1^T^ showed 3-CB utilization when growing with pyruvate. In contrast, HP culture did not use pyruvate, nor H_2_/CO_2_ for dehalogenation of 3-CB. Additionally, HP culture was also unable to dehalogenate 2-CB or 3-BB with either palmitate or H_2_/CO_2_ (Table 3). This is in line with the genomic analysis of the “*Ca.* Desulfomonile palmitatoxidans” sequences from HP culture. Genes encoding reductive dehalogenases have a conserved operon structure that consist of rdhA, coding for the catalytic subunit RdhA; rdhB, coding for a small putative membrane anchor; and a variable set of accessory genes coding for proteins involved in regulation and maturation of reductive dehalogenases (Lu et al., 2015). None of these genes were found in the metagenome. Therefore, combined with our physiological experiments that did not show any dehalogenation activity, it can be concluded that “*Ca.* Desulfomonile palmitatoxidans” lacks the potential for organohalide respiration.

Previous work suggested that *D. tiedjei* strain DCB-1^T^ has genomic potential to grow syntrophically (Worm et al., 2014). In our experiments, *M. formicicium* was chosen as the hydrogenotrophic methanogenic partner for the assessment of potential syntrophic relationships between *Desulfomonile* species. *M. formicicium* was shown as a syntrophic partner of LCFA-degrading bacteria, such as, e.g., *Syntrophomonas zehnderi* (Sousa et al., 2007c). Additionally, organisms assigned to *Methanobacterium* genus, most closely related to *M. formicicum*, are frequently present and are generally abundant in complex microbial communities degrading LCFA (Sousa et al., 2007a; Salvador et al., 2013, 2019; Cavaleiro et al., 2016). However, when HP culture or *D. tiedjei* strain DCB-1^T^ were incubated with *M. formicicum* and palmitate as electron donor, no methane or SCFA were produced after more than 137 days. These results suggest that LCFA conversion by the tested *Desulfomonile* members does not occur in syntrophy with hydrogenotrophic methanogens.

## Conclusion

We report the ability of *Desulfomonile* species to grow on MCFA and LCFA with sulfate as electron acceptor as a new physiological property of these bacteria. We also show that HP culture and *D. tiedjei* strain DCB-1^T^ cannot grow with palmitate coupled to organohalide respiration or syntrophically with *M. formicicum*. Since the *Desulfomonile* strain in HP culture could not be obtained in pure culture, a “*Candidatus*” taxon as a novel species belonging to *Desulfomonile* genus is proposed: “*Candidatus* Desulfomonile palmitatoxidans” palmitatoxidans (pal.mi.tat.o’xi.dans. N.L. masc. n. palmitas, -atis palmitate; N.L. v. oxido to make acid, oxidize; N.L. part. adj. palmitatoxidans oxidizing palmitate).

As a consequence of the newly reported characteristics, the description of the genus *Desulfomonile* by DeWeerd et al. (1990) has been emended, considering the following modifications: bacteria belonging to this genus are sulfate-reducing LCFA-degrading organisms and organohalide respiration is not an obligate characterizing feature of *Desulfomonile* genus.

This work gives new insights on LCFA-degrading microbial communities coupled to sulfate reduction.

## Data Availability Statement

The datasets generated for this study can be found in the European Nucleotide Archive (ENA)–LS453291 (https://www.ebi.ac.uk/ena/browser/view/LS453291), PRJEB26656 (https://www.ebi.ac.uk/ena/browser/view/PRJEB26656), PRJEB35900 (https://www.ebi.ac.uk/ena/browser/view/PRJEB35900).

## Author Contributions

AJC, DS, SA, and MA proposed and designed the study. AJC, DS, and SA provided guidance to JA, AFS, ARC, YZ, BN and streamlined communication between the different labs. JA, AFS, and YZ performed the initial enrichments, physiological characterization of HP culture, and assays with *Desulfomonile tiedjei*. ARC performed the enrichments and hydrocarbons analysis. BN and AFS performed the genomic analysis of HP culture and *D. tiedjei*. JA drafted the manuscript and the other authors participated in data interpretation as well as revisions of the final manuscript. All authors read and gave approval for publication of the manuscript.

## Conflict of Interest

The authors declare that the research was conducted in the absence of any commercial or financial relationships that could be construed as a potential conflict of interest.

## Footnotes

1 http://jspecies.ribohost.com/jspeciesws/
