# Supplementary material for: Long-Chain Fatty Acids Degradation by Desulfomonile Species and Proposal of “Candidatus Desulfomonile Palmitatoxidans”
Source: Front Microbiol. 2020 Dec 17;11:539604. doi: 10.3389/fmicb.2020.539604 (PMC7773648; doi:10.3389/fmicb.2020.539604)
Supplement: Supplementary file 4 [file Image_1.PDF]

Supplementary Material

Supplementary Figure 1.

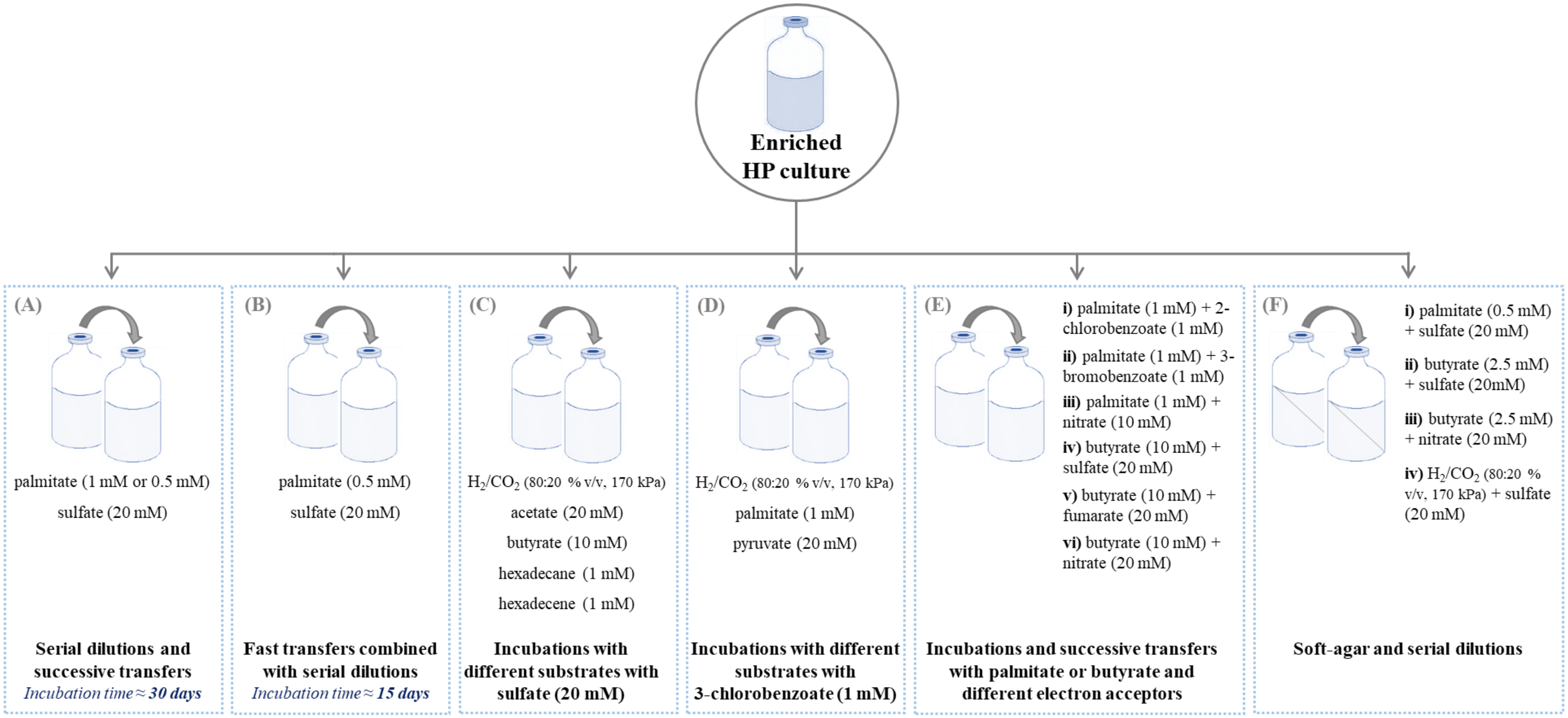

Supplementary Figure 1 – Scheme of the experimental procedures performed for the physiological characterization of HP culture. 120 mL serum bottles were used (50 mL liquid + 70 mL headspace); each transfer was done with 10 % (v/v) inoculum.
